# An international survey of pain in adolescents

**DOI:** 10.1186/1471-2458-14-447

**Published:** 2014-05-13

**Authors:** Michael Steven Swain, Nicholas Henschke, Steven James Kamper, Inese Gobina, Veronika Ottová-Jordan, Christopher Gerard Maher

**Affiliations:** 1 Department of Chiropractic, Faculty of Science, Macquarie University, Sydney 2109, Australia; 2 Musculoskeletal Division, The George Institute for Global Health, Sydney Medical School, University of Sydney, Sydney, Australia; 3 Institute of Public Health, University of Heidelberg, Heidelberg, Germany; 4 Department of Epidemiology and Biostatistics, EMGO + Institute, VU University Medical Center, Amsterdam, The Netherlands; 5 Department of Public Health and Epidemiology, Riga Stradinš University, Riga, Latvia; 6 Department of Child and Adolescent Psychiatry, Psychotherapy, and Psychosomatics, Research Unit Child Public Health, University Medical Center Hamburg-Eppendorf, Hamburg, Germany

**Keywords:** Pain, Adolescent, Prevalence, Epidemiology, World Health Organisation, HBSC

## Abstract

**Background:**

A common belief is that pain is uncommon and short lived in adolescents. However, the burden of pain in adolescents is unclear because of limitations in previous research. The aim of this study is to estimate the prevalence of headache, stomach-ache and backache in adolescents and to explore the extent to which these three forms of pain coexist based upon a representative sample of adolescents from 28 countries.

**Methods:**

Data were analysed from three consecutive waves (1997/98, 2001/02 and 2005/06) of the Health Behavior in School-aged Children: WHO Collaborative Cross-National survey (HBSC). Prevalence estimates are based upon adolescents who reported experiencing headache, stomach-ache or backache at least monthly for the last 6 months.

**Results:**

There were a total of 404,206 participants with a mean (±SD) age of 13.6 (±1.7) years (range 9.8 to 17.3 years). The prevalence of headache was 54.1%, stomach-ache 49.8%, backache 37%, and at least one of the three pains 74.4%. Girls had a higher prevalence of the three pains than boys and the prevalence of pain increased with age. Headache, stomach-ache and backache frequently coexist, for example, of those with headache: 21.2% had headache alone, 31% suffered from both headache and stomach-ache, 12.1% suffered from backache and headache, and 35.7% had all three pains.

**Conclusions:**

Somatic pain is very common in adolescents, more often coexisting than occurring in isolation. Our data supports the need for further research to improve the understanding of these pains in adolescents.

## Background

Adolescence marks the transition from childhood to adult life. Pain during adolescence is an important predictor of future pain [1-3]. A Danish twins study [4] found adolescents with persistent low back pain were 3.5 times more likely to have low back pain in adulthood. Co-occurrence of low back pain and headache in adolescence further increases the risk of developing future pain which draws attention to the significance of multiple pains [4].

Similar to adults, substantial economic costs can be attributed to pain in adolescents by way of direct medical costs, parental work absence and childcare expenses [5,6]. In adulthood the estimated cost of pain-related lost productivity time is $61.2 billon a year in the United States [7] with headache and back pain the leading contributors to this cost. In Europe the total cost of headache alone is estimated to exceed €20 billion per year [8]. Given the apparent link between adolescent pain and pain in adult life, steps to better understand and prevent adolescent pain are appropriate. An important first step in public health management is to identify the extent of the problem.

A number of systematic literature reviews have previously investigated the prevalence of headache [9,10], abdominal pain [10] and back pain [10,11] in children and adolescents. However, meaningful synthesis of the research in this area is hampered by the poor quality of the original studies. King and colleagues’ review [10] noted a considerable number of relatively small studies that yield imprecise estimates of prevalence, which are inadequate to make inferences at a global level. Between the studies there are large differences in the age range (children as young as 2 and as old as 18) and different instruments used to measure pain. Not surprisingly King et al’s review noted that the estimates of chronic pain prevalence vary substantially between studies (headache 8–83%; abdominal pain 4–53% and back pain: 14–24%); and there were inconsistent conclusions on the effect of age, region, psychosocial, and demographic factors on pain prevalence.

The study of prevalence from large, generalisable samples is critical in epidemiology and a paucity of such studies exists with regard to pain in adolescence. The World Health Organisation (WHO) monitors the health and behaviour of school-aged children via a survey conducted every four years [12,13] which enables the exploration of adolescent pain from a more global perspective. This dataset has advantages over previous studies in this area because it is derived from a large multi-national study using standardised methods of data collection. The ‘Health Behaviour in School-aged Children: WHO Collaborative Cross-National survey (HBSC)’ dataset has advantages over previous studies in this area because it is derived from a large multi-national study across Europe, North America, and Israel, using standardised methods of data collection. The purpose of the current study is to estimate the prevalence of headache, stomach-ache and backache in adolescents as well as explore the extent to which these pain conditions coexist using this data.

## Methods

### Study design and sample

Data were obtained from three consecutive waves (1997/98, 2001/02 and 2005/06) of the ‘Health Behaviour in School-aged Children: WHO Collaborative Cross-National Survey (HBSC)’. The HBSC research network is an international alliance of researchers that conduct four-yearly cross-national surveys. Data is collected from 11-, 13- and 15-year-olds regarding their health, well-being, social environments and health behaviours. A standardised research protocol has been developed by the HBSC research network for the purpose of securing valid, reliable, and comparable data.

The HBSC study design, methods and data collection dates have been described in detail elsewhere [14-16]. Three age groups – mean of 11.5, 13.5 and 15.5 years – are sampled via administration of surveys within school classes. For the majority of participating countries a nationally representative sample is drawn. The primary sampling unit is the school class or, where a sample frame of classes is not available, the whole school. In the latter circumstances sampling is performed across school grades to account for students that have been advanced or held back. Cluster sampling is therefore used in which the primary sampling unit is the class (or school) rather than the individual student. The desired sample size for each age group is 1500 students per country (750 per gender). Once data is collected from the participating countries files are exported to the HBSC data banks where data is checked and cleaned in accordance to strict criteria. A list of participating researchers, countries and select reports can be found at http://www.hbsc.org.

### Instrument and variables

Data from 28 countries across Europe, North America and Israel were extracted for this study. In Belgium separate surveys were conducted for Flemish and French speaking regions. Respondent demographics (gender, age and country) and data from the HBSC symptoms checklist (HBSC-SCL) were accessed. Responses to questions pertaining to headache, stomach-ache and backache were extracted for evaluation. The frequency of the respective pains was listed as a single multipart question: “In the last 6 months, how often have you had the following?” a list of symptoms included: headache, stomach-ache and backache. For each type of pain, respondents were required to specify the frequency of pain in the last six months on a five point scale: (1) “about every day”; (2) “more than once a week”; (3) “about every week”; (4) “about every month”; or (5) “rarely or never”. No details regarding the duration and intensity of somatic pain were available. The HBSC-SCL enables comparable assessment of pain across countries, age groups and genders [17].

### Data analysis

The prevalence of headache, stomach-ache and backache was estimated by analysing the combined data from the 1997/98, 2001/02 and 2005/06 survey waves. Prevalence estimates are based upon adolescents who reported experiencing headache, stomach-ache or backache at least every month for the last 6 months. Prevalence rates were calculated and then plotted using SigmaPlot version 12. The extent to which the three forms of pain coexist in adolescents was explored by constructing frequency distribution tables and cross-tabulation using SPSS version 20. Membership of the clusters of coexisting pains was illustrated using a three set area-proportional Venn diagram using an applet based on the method described by Chow and Rogers [18]. Univariate logistic regression models were constructed to investigate the odds of experiencing an individual pain type which also coexisted with another pain type. These were carried out using Statistical Analysis System (SAS) version 9.3.

## Results

Data from a total of 404,206 adolescents in 28 countries were available for analysis. Individual participants’ age ranged from 9.8 years to 17.3 years. For 11-, 13-, and 15-year age groups the mean age of respondents was 11.6 years, 13.6 years and 15.6 years respectively. There were slightly more girls (51.2%) than boys (48.8%) and the three waves were of similar size (Table 1).

**Table 1:** Descriptive statistics of participants

**Age**	**Mean: 13.6 years (SD: 1.7 years)**		
		**n**	**%**
Gender	Boy	197094	(48.8%)
	Girl	207112	(51.2%)
Country	Austria	13636	(3.4%)
	Belgium - Flemish	15424	(3.8%)
	Belgium - French	11304	(2.8%)
	Canada	16858	(4.2%)
	Czech republic	13497	(3.3%)
	Denmark	15479	(3.8%)
	England	17237	(4.3%)
	Estonia	10360	(2.6%)
	Finland	15501	(3.8%)
	France	19473	(4.8%)
	Germany	17716	(4.4%)
	Greece	11821	(2.9%)
	Greenland	3905	(1.0%)
	Hungary	11305	(2.8%)
	Israel	16401	(4.1%)
	Latvia	11501	(2.9%)
	Lithuania	15790	(3.9%)
	Norway	14760	(3.7%)
	Poland	16733	(4.1%)
	Portugal	10580	(2.6%)
	Rep. of Ireland	12163	(3.0%)
	Russia	20265	(5.0%)
	Scotland	16226	(4.0%)
	^*^Slovak republic	7671	(1.9%)
	^*^Spain	14718	(3.6%)
	Sweden	12143	(3.0%)
	Switzerland	14820	(3.7%)
	USA	14086	(3.5%)
	Wales	12833	(3.2%)
Wave	1997/98	122386	(30.3%)
	2001/02	135067	(33.4%)
	2005/06	146756	(36.3%)

(^*^Waves and countries without HBSC-SCL data: Spain 1997/98, Slovak republic 2001/02).

**Figure 1:**
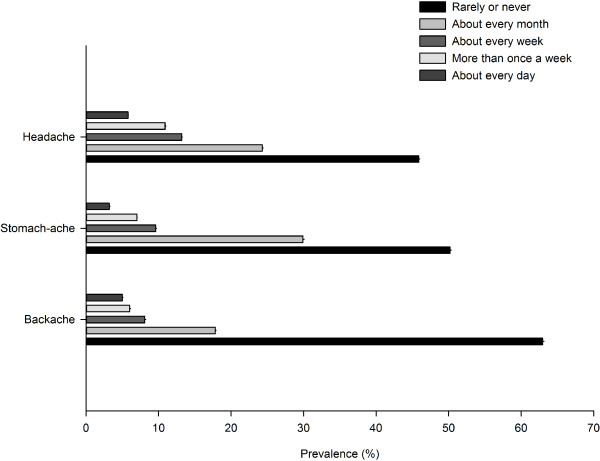
**Prevalence of headache, stomach-ache and backache by frequency of occurrence.** (Excluding adolescents who did not state: headache n = 5620, 1.4%; stomach-ache n = 6412, 1.6% and backache n = 7142, 1.8%).

Headache was the most prevalent of the three pain conditions in adolescents. The percentage of adolescents (95% confidence interval) who reported a headache monthly or more frequently was 54.1% (54.0% to 54.3%), stomach-ache was 49.8% (49.6% to 49.9%) and backache was 37% (36.8% to 37.1%). Figure 1 shows the frequency distribution of somatic pain in adolescents. The prevalence of headache, stomach-ache and backache stratified by country is presented as supplemental information (Additional file 1: Figure S1, Additional file 2: Figure S2 and Additional file 3: Figure S3). There was some variation in pain prevalence across the 28 countries, but in no countries were any of these three pains uncommon. The lowest pain prevalence was stomach-ache in Portuguese adolescents which affected 22.8% (22.0% to 23.6%).

**Figure 2:**
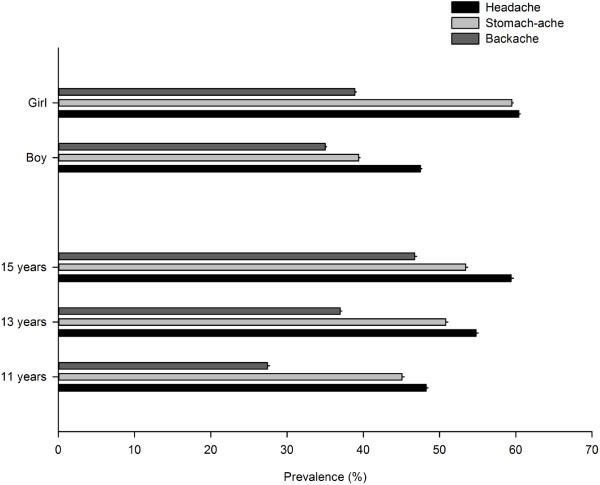
**Prevalence of headache, stomach-ache and backache by gender and age-group.** (Excluding adolescents who did not state: gender*headache n = 5620, 1.4%; gender*stomach-ache n = 6412, 1.6% and gender*backache n = 7142, 1.8%; age-group*headache n = 7590, 1.9%; age-group*stomach-ache n = 9096, 2.3%; age-group*backache n = 8378, 2.1%).

The three pains were more prevalent in girls and older adolescents (Figure 2). The prevalence in girls vs. boys was: headache 60.4% (60.1% to 60.6%) vs. 47.5% (47.3% to 47.7%); stomach-ache 59.5% (59.3% to 59.7%) vs. 39.4% (39.2% to 39.6%), and backache 38.9% (38.7% to 39.1%) vs. 35.0% (34.8% to 35.2%). The increase in prevalence from 11 to 13 to 15 years for headache was 48.3% (48.0% to 48.5%), 54.8% (54.5% to 55.1%) and 59.4% (59.1% to 59.7%); for stomach-ache was 45.1% (44.8% to 45.4%), 50.8% (50.5% to 51.1%) and 53.4% (53.2% to 53.7%); and for backache 27.4% (27.2% to 27.7%), 37.0% (36.7% to 37.2%) and 46.7% (46.5% to 47.0%).

The prevalence of having at least one of the three somatic pains was 74.4% (74.3% to 74.6%), with 47.3% (47.1% to 47.4%) of adolescents reporting two or more of the three pain conditions (Table 2). Girls experienced multiple pains more frequently than boys and multiple pains became more prevalent as age increased across adolescence. The prevalence of multiple pains stratified by gender and age is presented as supplemental information (Additional file 4: Figure S4).

**Table 2:** Coexistence of somatic pain in adolescents

**Number of somatic pains**	**Frequency (n)**	**Percent (%)**	**Cumulative percent (%)**
1	107451	27.1	27.1
2	110792	28.0	55.1
3	76475	19.3	74.4
0	101253	25.6	100.0
Total	395971	100.0	

(Excluding 8235 adolescents whose pain frequency was not stated).

**Figure 3:**
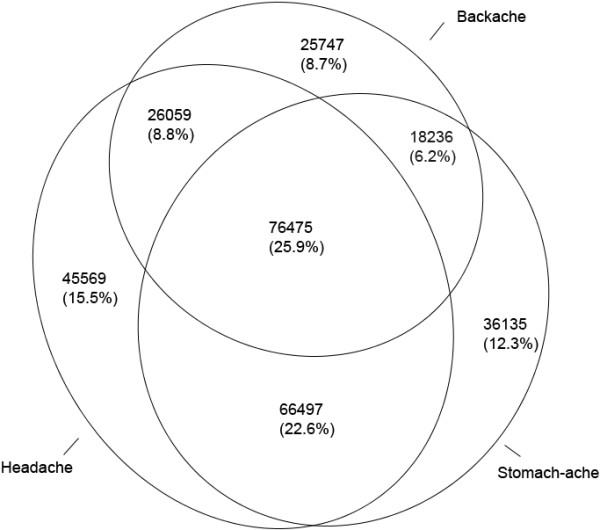
**Proportional Venn diagram representing the coexistence of pain in adolescents.** (Excluding 8235 adolescents whose pain frequency was not stated).

Figure 3 proportionally represents the extent to which the three pains coexisted in adolescents. It can be seen that each of the three pains commonly coexist with one or both of the other pain conditions. For example, of the adolescents with headache: 21.2% (21.1% to 21.4%) had headache alone, 31.0% (30.8% to 31.2%) also suffered stomach-ache, 12.1% (12% to 12.3%) suffered from backache and headache, and 35.7% (35.4% to 35.8%) had all three pains. Univariate logistic modelling found adolescents with pain (headache, stomach-ache or backache) were at increased odds of experiencing coexisting pain. This was highest for headache and stomach-ache OR = 4.7 (4.6 to 4.7), followed by headache and backache OR = 2.9 (2.8 to 2.9) and stomach-ache and backache OR = 2.6 (2.6 to 2.7).

## Discussion

Almost three-quarters of adolescents experience headache, stomach-ache or backache at least monthly. These pain conditions commonly coexist and are more prevalent in girls and older adolescents. While there was some variation in pain prevalence across the 28 countries there were no countries where these three pains were uncommon.

Our study is substantially larger than any previous study of the prevalence of pain in adolescents. It provides robust estimates of prevalence and coexistence of pain as it draws from a large, multi-national and representative sample of adolescents and makes use of the standardised survey methods employed by the HBSC research network. These methods minimise sampling bias and enable extrapolation of the results across Europe, North America and Israel. An important feature of the study is that it provides information on the prevalence of each of the three types of pain separately as well as in combination.

The HBSC Symptoms Check List (HBSC-SCL) was primary developed for measuring the subjective experience of health and in this study it quantifies the subjective experience of pain among adolescents regardless of the cause. A limitation of relying upon a brief instrument like this is that it does not provide a precise medical diagnosis for each adolescent’s pain or provide greater qualitative information on the pain experience. Given the broad nature of the pain measure, it is likely that physical ailments (such as sports injuries, menstrual issues and menstrual migraine) were among the causes of pain in this study. Having established the scale of the problem in this study we would encourage additional studies to further characterise these problems in adolescents.

Traditionally back pain is considered a condition of middle age and is regarded as being uncommon and/or short lived in adolescents. Reflecting this view current clinical practice guidelines specify that back pain in those younger than 20 years is a ‘red flag’ which should alert clinicians to the possibility of serious spinal pathology [19,20]. Further investigation via imaging and laboratory testing is then recommended. Our finding of a high prevalence of backache in adolescents questions the clinical utility of ‘age of onset <20 years’ as red flag to screen for serious disease [21]. Additionally many clinical practice guidelines explicitly state that their treatment recommendations only apply to adults [22]. Clinical practice guidelines should be reviewed to consider the implications of the high prevalence of pain in adolescents.

Our estimates of the prevalence of headache, stomach-ache and backache are at the upper bounds or higher than the wide range of previous estimates reported in King and colleagues’ review of chronic pain [10]. These differences may be explained in part by differences in ages of the young people studied, case definition, and recall period [23]. The way in which different types of somatic pain are defined contributes to variations in previous prevalence estimates. A study [24] of low back pain in British school-children (11-14 years, girls 53.9%) illustrates the effect of a different operational definition. Directed by a pain diagram and the severity measure ‘for one day or longer in the prior month’, adolescents reported the prevalence of back pain as 26%, which is substantially less than the 37.0% backache estimate obtained for England in this analysis. Moreover, adolescence is defined by the WHO as the period between 10 and 19 years [25]. The HBSC study methods encompass a broad age range but ensure that a minimum of 95% of the eligible target population falls within the sample frame 11-15 years [12]. Given that the prevalence of somatic pain in children and adolescents increases with age it is reasonable to suggest that disparities in pain prevalence estimates may be explained by the variability in the age distribution of previous studies. For example, a Swedish study [26] reported the monthly prevalence of headache in children (7-16 years, girls 48.6%) to be 26%, which is substantially lower than this study’s estimate of 63.1% from Sweden. The difference is likely due to the fact that 20% of the sample was aged below 11 years. A one-month time period for reporting prevalence was used in this study on the basis of recently published consensus [27] and empirically-based [28] recommendations in the field of back pain. Opportunity exists for a consensus approach to standardise important definitional components of paediatric somatic pain including the frequency and duration of pain and the age distribution in sampling strategies.

Our study found somatic pain in adolescents most commonly occurs in multiple-pain form. In particular young people had the greatest odds of coexisting pain when they experienced headache and stomach-ache, which appears to align with the prevailing knowledge [29]. Contrary to a previous study [29] we found girls have a higher prevalence of multiple-pains than boys and the prevalence of multiple-pains increases with age. Given the high prevalence of individual pains it is likely that some coexistence of individual pains will occur despite unrelated cause. Several potential physical, behavioural and mental developmental changes that coincide with pubertal development have been hypothesised to explain the age and gender differences that were observed in this study [11,30,31]. LeResche et al. [31] found both the prevalence of one pain condition and the prevalence of two or more pain conditions increased with increasing physical maturity, which may explain the significant increase in the pain prevalence with age in our study. Very few studies have described the extent to which somatic pains coexist in adolescents and as a consequence there is a paucity of knowledge in this field.

Suffering and developmental consequences are important actual and potential implications of somatic pain in adolescence. Somatic pain has been associated with anxiety and depression as well as school absenteeism and poor quality of life [5]. The direct cost of health care is likely to already be apparent given the relationship between subjective health complaints and high medicine use in adolescents [32]. Somatic pain during adolescence is associated with re-occurrence later in life [1-4] and it appears that some groups of children are predisposed to ongoing pain-related problems, including work disruption, into adulthood [33]. Given that the majority of sick leave in adults is due to somatic pains [34], prevention and management of these problems in adolescence could conceivably have an important impact on disease burden in adults.

## Conclusions

Our research has clearly established that headache, stomach-ache and backache are very common in adolescents and these pains more often coexist than occur in isolation. Somatic pain affects the health and well-being of adolescents in several countries across Europe and North America and as such poses a substantial public health challenge. However, research into the health of young people is recognised as a neglected priority in global health [35] and this is certainly the case in the pain field where there is an incomplete understanding of the epidemiology, mechanisms and management of these pains in adolescents. In regards to pain, large differences in prevalence exist across gender and age in adolescents. These findings are useful as they identify that girls are more likely to experience individual and multiple pains. Moreover, young people during late adolescence are commonly afflicted by multiple pains. Longitudinal investigations that coincide with the onset of pubertal development are now required to appropriately establish fundamental risk factors and mechanisms for adolescent pain. Once established, an evidence-based approached to prevention and intervention strategies can be explored in the interest of public health.

## Abbreviations

HBSC: Health Behaviour in School-aged Children; HBSC-SCL: Health Behaviour in School-aged Children – symptoms checklist; WHO: World Health Organisation.

## Competing interests

The authors have no competing interests to disclose.

## Authors’ contributions

MSS, NH and CGM conceptualised and designed the analyses. NH, IG and VO facilitated the data acquisition, MS conducted the analyses. All authors interpreted the data, critically reviewed and revised the manuscript. All authors approved the final manuscript and MS is the guarantor.

## Pre-publication history

The pre-publication history for this paper can be accessed here:

http://www.biomedcentral.com/1471-2458/14/447/prepub

## Supplementary Material

Additional file 1: Figure S1 Prevalence of headache in adolescents by country. (Excluding 5620 adolescents whose headache frequency was not stated). Click here for file

Additional file 2: Figure S2 Prevalence of stomach-ache in adolescents by country. (Excluding 6412 adolescents whose stomach-ache frequency was not stated). Click here for file

Additional file 3: Figure S3 Prevalence of backache in adolescents by country. (Excluding 7142 adolescents whose backache frequency was not stated). Click here for file

Additional file 4: Figure S4 The prevalence of multiple somatic pains stratified by (a) gender and (b) age. (Excluding 8235 adolescents whose pain frequency was not stated). Click here for file
