# Supplementary figures and images for: An international survey of pain in adolescents
Source: BMC Public Health. 2014 May 13;14:447. doi: 10.1186/1471-2458-14-447 (PMC4046513; doi:10.1186/1471-2458-14-447)

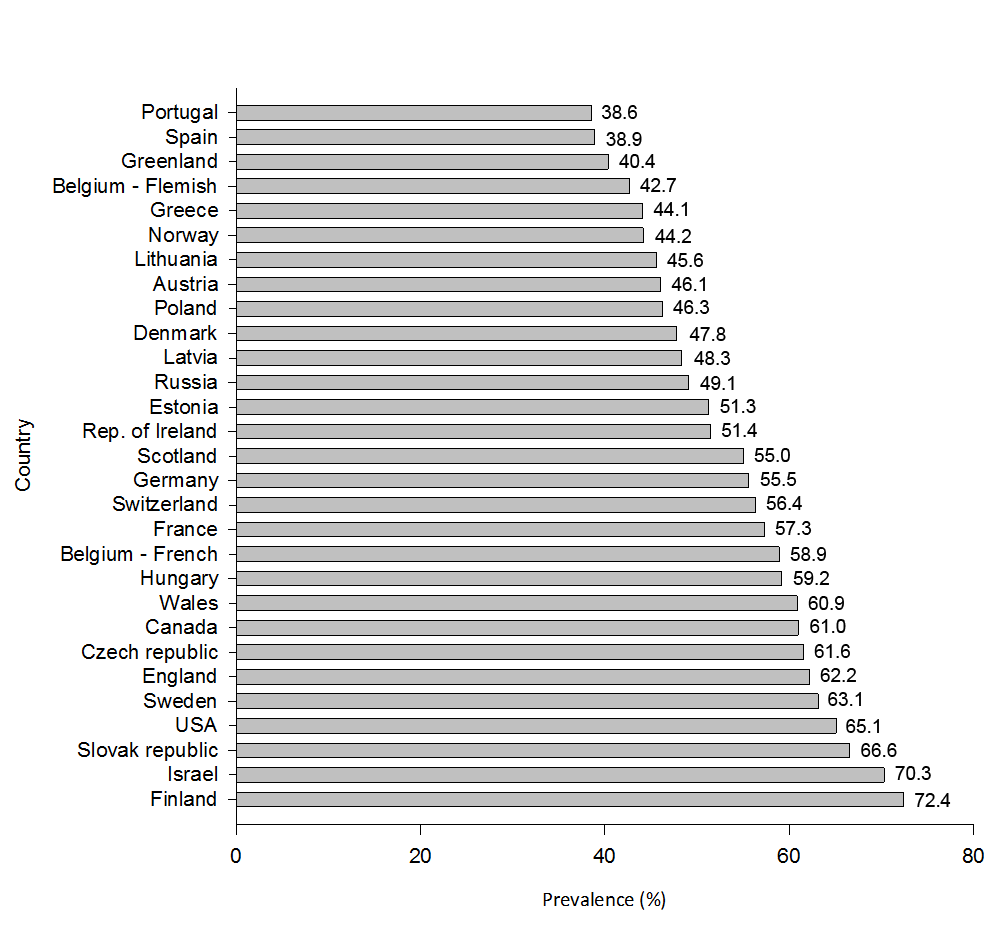

Supplement: Additional file 1: Figure S1 — Prevalence of headache in adolescents by country. (Excluding 5620 adolescents whose headache frequency was not stated). [file 1471-2458-14-447-S1.tiff]

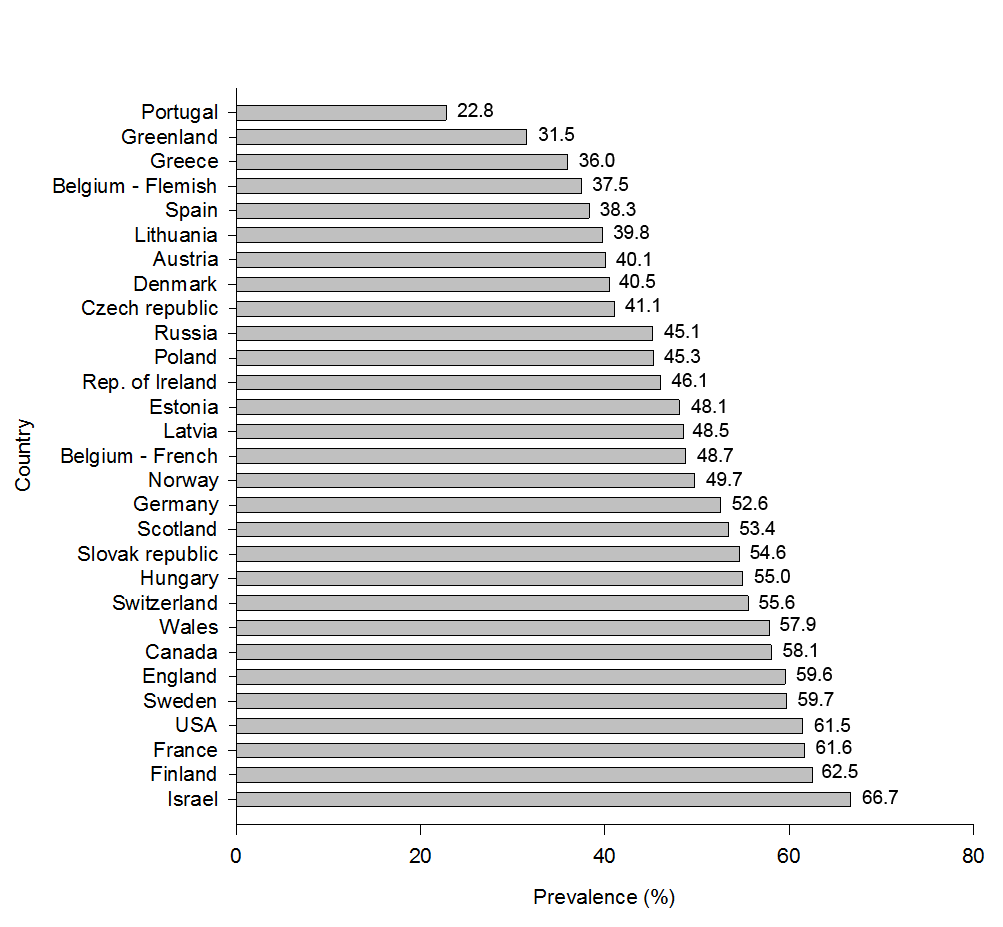

Supplement: Additional file 2: Figure S2 — Prevalence of stomach-ache in adolescents by country. (Excluding 6412 adolescents whose stomach-ache frequency was not stated). [file 1471-2458-14-447-S2.tiff]

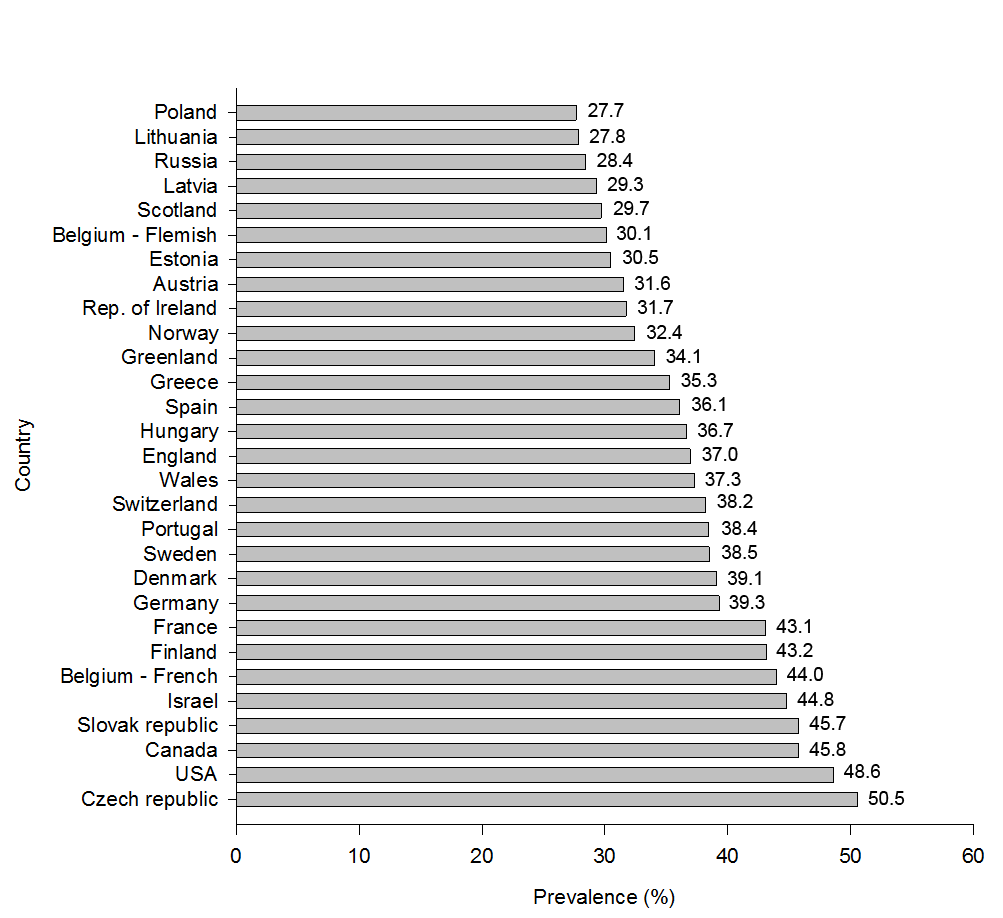

Supplement: Additional file 3: Figure S3 — Prevalence of backache in adolescents by country. (Excluding 7142 adolescents whose backache frequency was not stated). [file 1471-2458-14-447-S3.tiff]

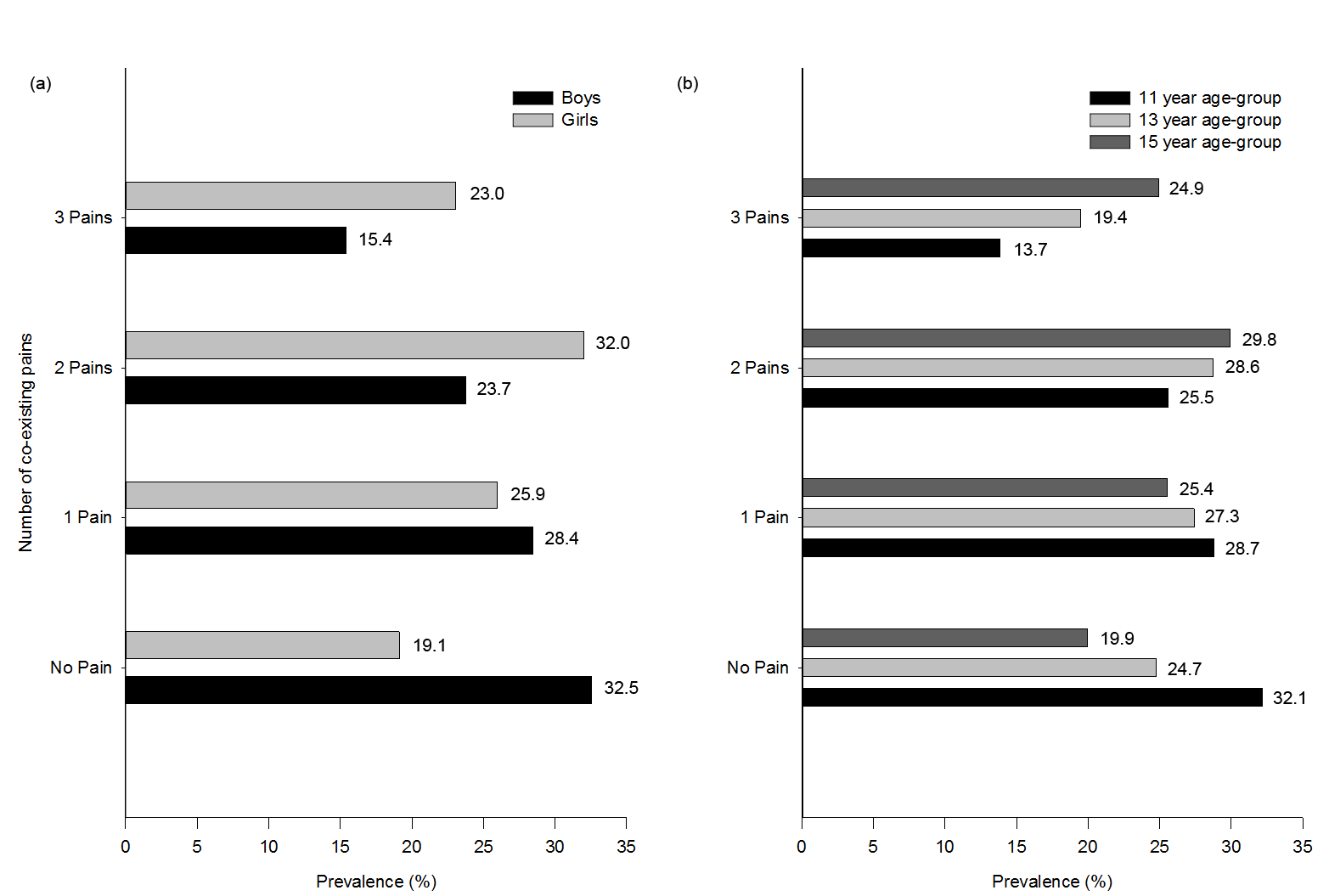

Supplement: Additional file 4: Figure S4 — The prevalence of multiple somatic pains stratified by (a) gender and (b) age. (Excluding 8235 adolescents whose pain frequency was not stated). [file 1471-2458-14-447-S4.tiff]
